# Contrast-Induced Type II Kounis Syndrome Complicated by a Cardiac Arrest Successfully Managed With Extracorporeal Membrane Oxygenation (ECMO): A Case Report

**DOI:** 10.7759/cureus.105111

**Published:** 2026-03-12

**Authors:** Bo Liao, Zhipeng Ding, Zhao Lin, Weixing Ge, Kun Zhang

**Affiliations:** 1 Department of Intensive Care Medicine, The Affiliated Jiangning Hospital with Nanjing Medical University, Nanjing, CHN

**Keywords:** acute coronary syndrome, allergic reaction, ecmo, extracorporeal membrane oxygenation, iodinated contrast, kounis syndrome

## Abstract

Kounis syndrome (KS) is a rare allergic-mediated acute coronary syndrome (ACS) characterized by coronary vasospasm, plaque rupture, or thrombosis. It is typically triggered by allergens such as medications, contrast agents, or environmental factors. We report the case of a 58-year-old man with hypertension who developed Type II KS after iodinated contrast administration for coronary evaluation. Shortly after contrast exposure, he experienced sudden hypotension, chest pain, and loss of consciousness, followed by cardiac arrest despite prompt resuscitation. Veno-arterial extracorporeal membrane oxygenation (VA-ECMO) and an intra-aortic balloon pump (IABP) were initiated for refractory hemodynamic instability. Coronary computed tomography angiography (CCTA) demonstrated atherosclerosis with 80% stenosis of the proximal left anterior descending artery (LAD), which, in the clinical context of contrast exposure and systemic allergic manifestations, supported the diagnosis of Type II KS. Over the following days, cardiac biomarkers declined, and ECMO was successfully weaned. Follow-up imaging confirmed adequate coronary perfusion without the need for percutaneous coronary intervention (PCI). The patient was discharged without neurological deficits at the three-month follow-up. This case underscores the importance of early recognition of KS, especially in patients with underlying coronary artery disease, and highlights the potential role of ECMO as rescue support in hemodynamically unstable presentations.

## Introduction

Kounis syndrome (KS) is an allergic form of acute coronary syndrome (ACS) that arises from mast cell activation in response to various triggers. It can present as coronary vasospasm, plaque destabilization, or stent thrombosis [1]. Reported triggers include food allergens, antibiotics, nonsteroidal anti-inflammatory drugs, anesthetics, contrast agents, and multiple environmental factors [2, 3]. KS can occur in individuals without pre-existing coronary artery disease (CAD) or exacerbate ischemia in those with underlying coronary pathology [1]. The syndrome, termed “allergic angina” or “allergic myocardial infarction” due to its pathophysiological basis, is classified into three types: Type I (vasospasm in normal coronary arteries), Type II (allergic ACS in patients with underlying coronary artery disease), and Type III (allergic stent thrombosis).

KS has been reported across a broad age spectrum but occurs most commonly in men aged 40-70 years [2, 4]. Estimates suggest a prevalence of 1.1%-3.4% [5], though the true rate may be higher because its presentation often mimics other cardiovascular emergencies, contributing to underdiagnosis.

The underlying mechanism involves mast cell degranulation and the release of mediators such as histamine, leukotrienes, and platelet-activating factor, which can provoke coronary vasospasm, enhance platelet aggregation, and contribute to plaque instability or thrombosis [6,7]. In severe cases, systemic vasodilation, hypotension, and arrhythmias can lead to circulatory collapse.

Although KS can be severe, optimal management strategies, particularly regarding the timing and selection of mechanical circulatory support in refractory shock or malignant arrhythmias, remain unclear. In such settings, veno-arterial extracorporeal membrane oxygenation (VA-ECMO) may provide a temporary bridge during potentially reversible coronary vasospasm or transient myocardial dysfunction. We report a case of contrast-induced Type II KS complicated by ventricular fibrillation (VF) and refractory cardiogenic shock, successfully treated with VA-ECMO and intra-aortic balloon pump (IABP). This case underscores the importance of early recognition and suggests that ECMO may serve as a rescue option in severe KS.

## Case presentation

A 58-year-old male patient (174 cm, 84 kg) with a history of hypertension presented to the emergency room with recurrent chest tightness. The baseline electrocardiogram (ECG) showed no ischemic changes, and coronary computed tomography angiography (CCTA) was scheduled to evaluate possible CAD. He had no known allergies, and pre-procedural laboratory tests were normal. He was not taking beta-blockers or other antiarrhythmic medications prior to admission.

**Figure 1:**
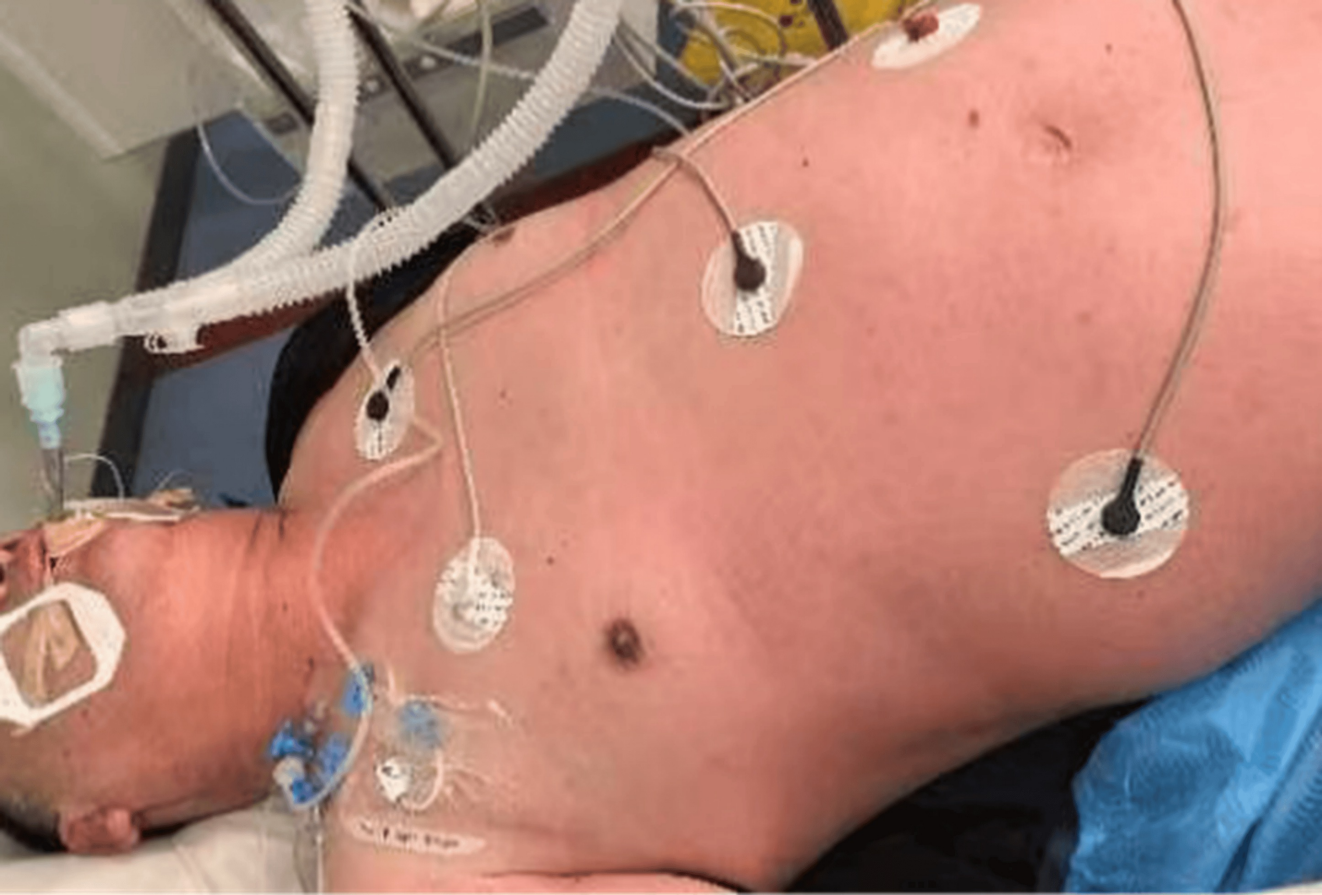
Diffuse facial and truncal flushing observed upon the patient's arrival at the emergency room.

Immediately after completion of the CCTA examination with iodinated contrast (iohexol), the patient developed acute stabbing chest pain and dizziness. Within one to two minutes, he collapsed and lost consciousness near the CT suite and was immediately transferred to the emergency room. Upon arrival (approximately three to four minutes after symptom onset), he exhibited diffuse facial and truncal flushing (Figure 1) and hypotension (non-invasive blood pressure 78/42 mmHg). Anaphylactic shock was suspected. Intramuscular epinephrine (0.5 mg) was administered approximately five minutes after initial symptom onset. Intravenous fluid resuscitation was initiated, and norepinephrine infusion was started at 0.3 μg/kg/min due to persistent hypotension. No corticosteroids or antihistamines were administered during the initial resuscitation. Due to persistent unconsciousness requiring airway protection, endotracheal intubation was performed in the emergency room approximately eight minutes after symptom onset.

**Figure 2:**
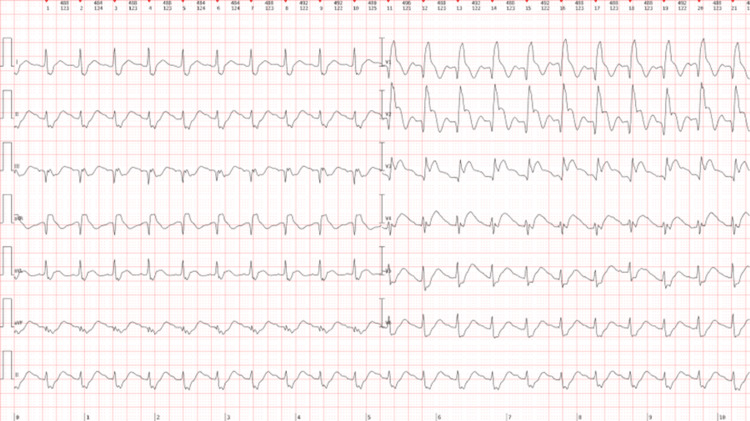
Electrocardiogram at the patient’s arrival in the emergency room, showing abnormal Q waves (III, aVF, and V1–V3), ST-segment elevation (aVL and V1–V4), reciprocal ST depression (II, III, and aVF), and right bundle branch block.

The ECG (Figure 2) showed abnormal Q waves (III, aVF, and V1-V3), ST-segment elevation (aVL and V1-V4), reciprocal ST depression (II, III, and aVF), and findings compatible with right bundle branch block. Although acute myocardial infarction was suspected, emergent coronary angiography was deferred due to profound hemodynamic instability and the high procedural risk in the immediate setting of suspected contrast-induced anaphylactic shock. The priority was circulatory stabilization. The patient was therefore transferred to the intensive care unit (ICU) about 30 minutes after initial symptom onset for ongoing resuscitation and advanced hemodynamic support.

At approximately 45 minutes after symptom onset, cardiac monitoring revealed polymorphic ventricular tachycardia that degenerated into VF with loss of carotid pulse. Recurrent VF occurred at 53 and 63 minutes. A total of three biphasic 200-J defibrillation shocks were delivered. The cumulative duration of cardiopulmonary resuscitation (CPR) was approximately 15 minutes. Intravenous epinephrine (1 mg every three to five minutes) and amiodarone (300 mg IV bolus as the first dose) were administered according to the Advanced Cardiovascular Life Support (ACLS) protocol [8]. Return of spontaneous circulation (ROSC) was achieved after each episode. Despite repeated ROSC, the patient remained profoundly hemodynamically unstable, with a mean arterial pressure of approximately 50 mmHg despite norepinephrine infusion up to 0.8 μg/kg/min. Transthoracic echocardiography (TTE) demonstrated an ejection fraction of 41%. Given recurrent ventricular fibrillation and refractory circulatory collapse, VA-ECMO was initiated via right femoral artery and vein cannulation (flow rate 3.0 L/min), followed by insertion of an IABP and implementation of targeted temperature management.

**Figure 3:**
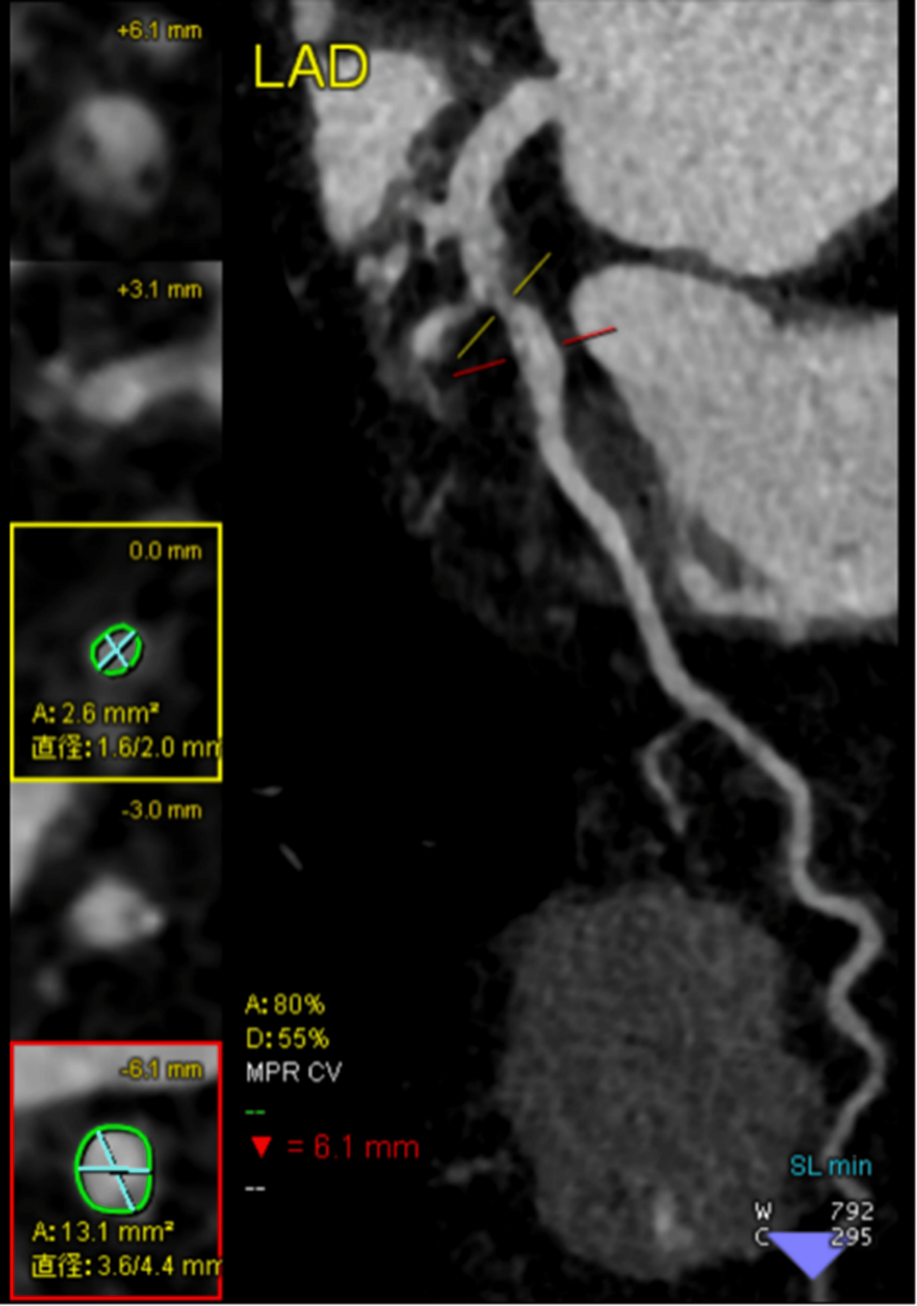
Left anterior descending artery (LAD) showing positive remodeling, punctate calcifications, and stenosis (80%) on coronary computed tomography angiography.

**Figure 4:**
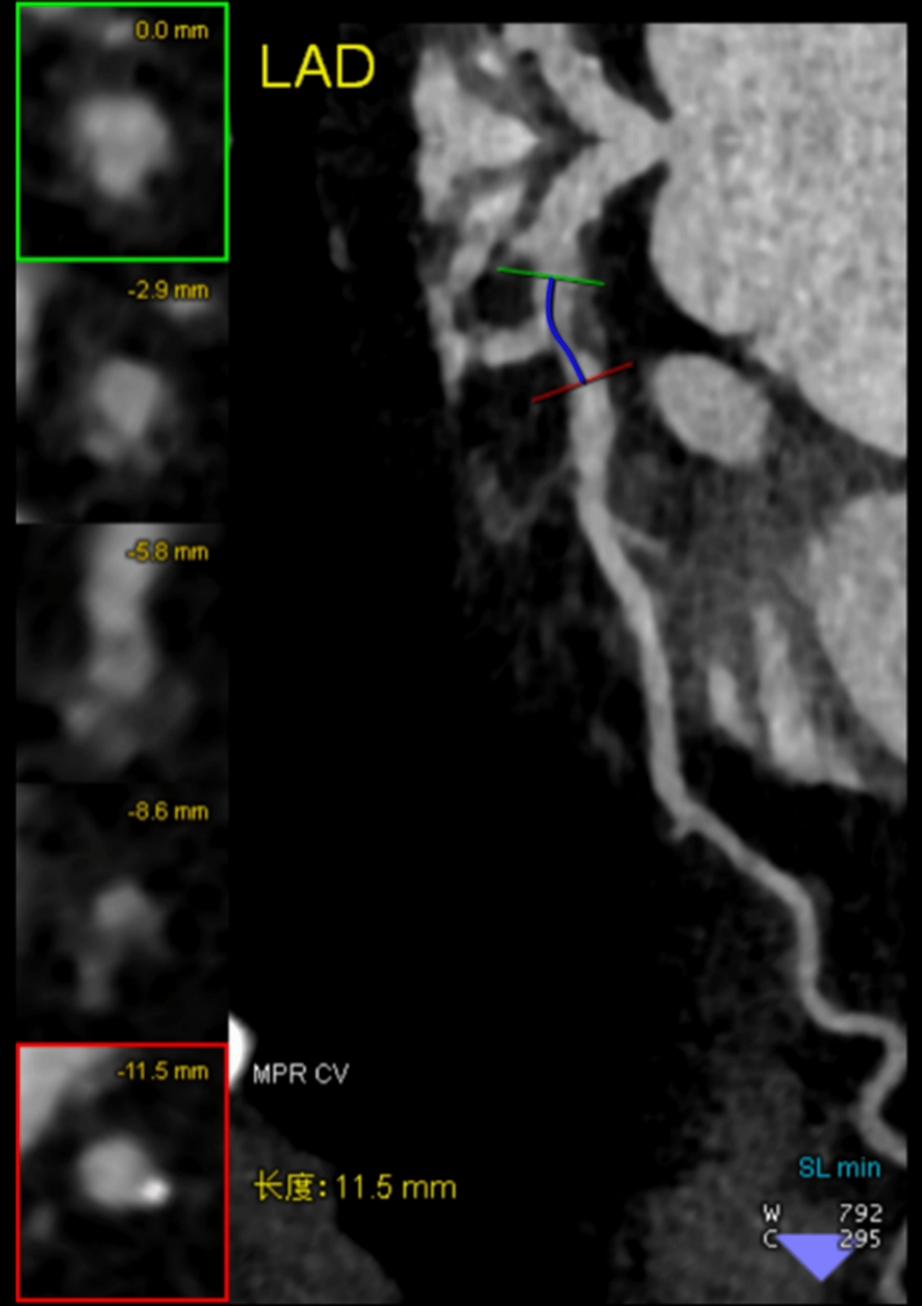
Stenosis (11.5 mm in length) of the left anterior descending artery (LAD) as seen on coronary computed tomography angiography.

CCTA performed prior to hemodynamic collapse demonstrated a mixed plaque in the proximal left anterior descending artery (LAD) with positive remodeling, punctate calcifications, and stenosis (80%, length: 11.5 mm) (Figures 3-4). This lesion was classified as Coronary Artery Disease-Reporting and Data System (CAD-RADS) 4A/high-risk plaque [9]. The mid-LAD also showed moderate stenosis and a deep myocardial bridge. Given the recent contrast-induced anaphylaxis and ongoing circulatory instability, percutaneous coronary intervention (PCI) was deferred in favor of conservative management, including anticoagulation, dual antiplatelet therapy (aspirin and clopidogrel), statin therapy, and coronary vasodilators. Cardiac biomarkers peaked within the first 24 hours and subsequently declined. By hospital day 3, hemodynamic status improved, and VA-ECMO was successfully weaned. The patient was extubated on day 5 and transferred to a specialized center on day 8.

At the referral center, intravascular ultrasound (IVUS) confirmed a fibrous-calcified plaque in the proximal-to-mid LAD with a minimum lumen area of 4.74 mm². The coronary flow reserve (fractional flow reserve (FFR) 0.92) indicated adequate perfusion, and PCI was not performed. The patient was discharged four days after transfer (hospital day 12 overall). At discharge, the patient was prescribed dual antiplatelet therapy (aspirin 100 mg daily and clopidogrel 75 mg daily), statin therapy (atorvastatin 40 mg daily), and beta-blocker therapy (metoprolol succinate 47.5 mg daily). An iodinated contrast allergy was documented in his medical records, and he was advised to avoid future exposure. Referral for formal allergy testing and evaluation was arranged. At the three-month follow-up, he remained asymptomatic without neurological deficits.

## Discussion

KS refers to the coexistence of acute coronary events and allergic reactions. In this case, the patient developed profound hypotension shortly after exposure to iodinated contrast, with coronary CTA revealing significant proximal LAD stenosis (80%), supporting the diagnosis of Type II KS. Despite standard treatment including epinephrine and fluid resuscitation, the patient continued to experience recurrent VF and circulatory collapse, requiring VA-ECMO and IABP. This highlights the need for heightened awareness of KS in patients with underlying CAD who experience severe allergic reactions and suggests that early hemodynamic support is crucial for improving prognosis.

The pathophysiology of KS involves mast cell activation and degranulation with the release of histamine, leukotrienes, and platelet-activating factor, promoting coronary vasospasm, platelet activation, and a pro-thrombotic state [6,7]. MRGPRX2-mediated pathways may also contribute to non-IgE-dependent mast cell activation [10,11]. Based on coronary anatomy and clinical presentation, KS is classified into three subtypes: Type I (vasospasm in normal coronaries), Type II (ischemia or infarction in pre-existing coronary artery disease), and Type III (stent thrombosis) [1]. This case fits Type II because the patient had both an allergic reaction and imaging-confirmed coronary stenosis, with myocardial ischemia temporally related to contrast exposure.

Diagnosis requires integrating allergic symptoms, cardiac ischemia, and imaging findings. Clinical manifestations range from flushing and nausea to hypotension, arrhythmias, or cardiac arrest [12]. ECG frequently shows ST-segment elevation or arrhythmias [4, 13, 14]. Cardiac biomarkers may remain normal in Type I but often rise in Types II and III [13, 14]. In the present case, cardiac biomarkers showed an early rise followed by a gradual decline, consistent with transient myocardial injury rather than ongoing coronary occlusion. Imaging is critical for evaluating the coronary substrate. Coronary CTA in this case demonstrated a high-risk proximal LAD plaque, while subsequent IVUS confirmed a fibrous-calcified lesion with a minimum lumen area of 4.74 mm². FFR (0.92) indicated preserved physiological flow, supporting the decision to defer PCI and reinforcing the diagnosis of Type II KS rather than primary plaque rupture.

Several alternative explanations should also be considered. Primary ACS due to plaque rupture was less likely because coronary CTA and IVUS demonstrated a stable fibrous-calcified plaque without evidence of rupture or thrombus, and FFR indicated preserved physiological flow. Demand ischemia secondary to anaphylactic shock may contribute to myocardial injury; however, the close temporal relationship between iodinated contrast exposure, systemic allergic manifestations, and ischemic ECG changes supports the diagnosis of Kounis syndrome. Epinephrine-induced ischemia is another possibility, but myocardial ischemia and ECG abnormalities occurred before substantial cumulative epinephrine exposure. Takotsubo syndrome and myocarditis were also considered, yet the presence of coronary stenosis together with the allergic trigger and dynamic ECG findings favored Type II KS.

Management of KS requires addressing both the allergic reaction and the associated coronary event. Standard ACS therapy, including antiplatelet agents, may be required in Type II and III disease [15]. Antihistamines and corticosteroids may relieve allergic symptoms [15]. Epinephrine remains the first-line treatment for anaphylaxis; however, its α-adrenergic vasoconstrictive effects may aggravate coronary spasm or trigger arrhythmias in KS [13]. Glucagon may be considered as an alternative, particularly in patients on β-blockers, due to its ability to increase intracellular cAMP [3,16]. Calcium channel blockers and nitrates can relieve vasospasm but should be used cautiously in hypotensive patients [13]. In Type III KS, stent thrombosis may require urgent revascularization [13,17].

ECMO in KS

In this case, TTE demonstrated reduced left ventricular ejection fraction, suggesting acute myocardial dysfunction potentially related to allergic-mediated coronary vasospasm and global hypoperfusion. Recovery of ventricular function in KS is influenced by allergen dose, individual susceptibility, and underlying coronary disease [18]. Patients with pre-existing cardiovascular conditions are particularly vulnerable to hemodynamic collapse [19]. Although evidence for ECMO use in KS remains limited and largely derived from case reports, several studies have reported successful application of ECMO in patients with refractory hypotension or cardiac arrest secondary to severe allergic reactions [20, 21]. Moreover, ECMO and extracorporeal CPR (ECPR) have gained increasing attention in contemporary resuscitation research as rescue strategies for refractory shock and cardiac arrest [22]. In the present case, VA-ECMO provided circulatory support after persistent VF and hemodynamic instability despite ACLS, allowing time for myocardial recovery. Anticoagulation during ECMO may also mitigate allergy-associated coronary thrombosis.

Limitations

This report has several limitations. First, as a single case report, causality cannot be definitively established. Second, confirmatory allergy testing was not performed during the acute phase, and therefore, the precise immunologic mechanism could not be fully characterized. Third, catecholamine administration during resuscitation may have contributed to myocardial ischemia or coronary vasospasm and cannot be completely excluded as a potential confounder. Finally, although coronary CTA and IVUS did not demonstrate plaque rupture or thrombus, the exact coronary mechanism, whether transient vasospasm, plaque destabilization, or a combination of both, cannot be definitively determined.

## Conclusions

This case illustrates that VA-ECMO may serve as an effective short-term rescue therapy for patients with Type II KS who develop refractory hypotension, malignant arrhythmias, or acute left ventricular dysfunction. Several clinical lessons emerge. First, KS should be suspected when acute coronary events occur shortly after exposure to contrast agents or other allergens in the presence of systemic allergic manifestations. Second, management requires balancing prompt treatment of anaphylaxis with awareness of potential coronary vasospasm and arrhythmia risk. Third, early multidisciplinary consideration of mechanical circulatory support, including VA-ECMO, may be life-saving in patients with refractory shock or cardiac arrest. Further studies are needed to clarify the optimal role of extracorporeal support in severe presentations of KS.
